# Research on irradiated food status and consumer acceptance: A Chinese perspective

**DOI:** 10.1002/fsn3.3511

**Published:** 2023-06-19

**Authors:** Ke Wang, Xinxin Pang, Zhengkui Zeng, Houhua Xiong, Jifu Du, Gang Li, Isaac Kwasi Baidoo

**Affiliations:** ^1^ School of Nuclear Technology and Chemistry & Biology and Hubei Key Laboratory of Radiation Chemistry and Functional Materials Hubei University of Science and Technology Xianning China; ^2^ China Isotope & Radiation Corporation Beijing China; ^3^ CNNC High Energy Equipment (Tianjin) Co., Ltd Tianjin China; ^4^ Nuclear Reactors Research Centre National Nuclear Research Institute Legon Ghana

**Keywords:** acceptance, consumer perception, irradiation food, nuclear technology

## Abstract

China is currently the world's largest producer of food irradiation. Despite the long‐standing (about 100 years) evidence supporting the safety of food irradiation, consumers’ acceptance of irradiated foods remains limited. This study aimed to investigate the development of food irradiation in China and identify the barriers that keep consumers away from irradiated foods. This was accomplished by exploring the relevant policies of food irradiation, the size and distribution of irradiation facilities in China, and analyzing their relationships between consumer characteristics and the acceptance of irradiated food. To achieve these objectives, we conducted an online survey of participants from Hubei, China (*N* = 264). The results reveal that irradiation facilities are mainly distributed in large coastal cities such as the Bohai Bay, the Yangtze River Delta, and the Greater Bay Area. Furthermore, the study identified that consumer’ acceptance of irradiated food is directly related to their level of understanding. Approximately 22% of the sampled consumers reported that they would not accept that they have consumed irradiated food and most of them (41%) stated that they would not purchase irradiated food if they were aware of buying irradiated food. Specifically, consumers expressed discomfort with consuming irradiated food under unknown circumstances. This trend is more prevalent among female, low‐educated, and older consumers, with 40% of the sampled population indicating that they would not buy irradiated food. Given the strong correlation between knowledge and acceptance of irradiated foods, the study suggests that policy reform should prioritize enhancing the understanding of irradiated food, particularly among female, low‐educated, and older consumers.

## Graphical abstract

This was accomplished by exploring the relevant policies of food irradiation, the size and distribution of irradiation facilities in China, and analyzing their relationships between consumer characteristics and the acceptance of irradiated food. At the same time, we identified that consumers' acceptance of irradiated food is directly proportional to their understanding: 22% of the sampled consumers will not accept that they have eaten irradiated food.Strong correlation between knowledgeable consumers and general acceptability of irradiated foods, we suggest that policy reform towards strengthening the understanding of irradiated food among female, low‐educated and older consumers

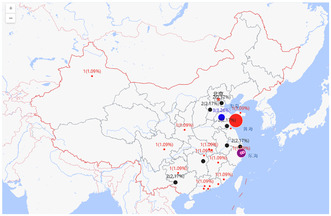

## 1 INTRODUCTION

Food pests, diseases, mildew, and among other factors have resulted in significant losses in the food industry. It is estimated that up to one‐third of the world's harvested agricultural produce is lost due to spoilage or pests before reaching consumers (Ashraf et al., 2019). Additionally, the poor hygienic quality of some locally produced foods has led to frequent outbreaks of food‐borne diseases, posing serious threats to public health and life safety. Food irradiation has proven to be a safe and efficient method to extend the shelf life of food, reduce spoilage, eliminate pests, and inactivate bacteria that cause food poisoning (Shi et al., 2020; Yoosefian et al., 2022). Many international organizations, such as the International Atomic Energy Agency (IAEA), Food and Agriculture Organization (FAO), the World Health Organization (WHO), and the Scientific Committee on Food of the European Commission, have concluded that food irradiated with suitable technologies is very safe and nutritionally acceptable (Arshad et al., 2020; Farkas & Mohácsi‐Farkas, 2011; Shahi et al., 2021).

As the largest and most populous country in the Asia‐Pacific region, and a major food producer and consumer, with 3 million tons of food consumed daily (Ministry of Justice of the People's Republic of China, 2022), China's food safety issues are not only related to the health and safety of consumers but also to the image of the government and the country, and to economic development and social stability. With the continuous improvement of food consumption requirements, food safety has become the focus of attention in China. Therefore, food irradiation technology has developed rapidly in China. Currently, China's irradiated foods, mainly consumed domestically, account for more than one‐third of the global total and include spices, dehydrated vegetables, and pickled peppers (The State Council Information Office of the People's Republic of China, 2022). The output of the irradiated processed foods industry in China contributes to 10% of the gross national products, making it an important pillar of the national economy (Dai Xiaofeng et al., 2018). China has approved 57 kinds of food suitable for irradiation in eight categories and has established a series of standards for food radiation processing. In 2016, China irradiated more than 500,000 tons of foods, making it the largest volume of processed foods in the world, including spices, garlic, grain, meat, and other products (Prakash, 2016).

Despite the history of more than 100 years of research on irradiated foods and the safety has been proved, consumers awareness and acceptance of irradiated foods remain low due to unfounded fears about nuclear technology, resulting in widespread consumer concerns about irradiated foods that vary across different regions of the world (Mostafavi et al., 2012). Based on these reasons, this study has analyzed the development of irradiated food and consumers’ understanding and acceptance of irradiated food from the perspective of China, aiming to provide a foundation for the healthy and rapid development of irradiated food in China (and the world).

## 2 REGULATORY DEVELOPMENT

Ionizing radiation has been extensively studied and used in food processing operations for public health and trade reasons since the 1920s, with systematic research and application beginning in the early 1950s. In October 1980, the Joint Expert Committee on Irradiated Foods of the Food and Agriculture Organization of the United Nations (FAO), the International Atomic Energy Agency (IAFA), and the World Health Organization (WHO) confirmed that irradiation at a dose below 10 kGy is safe for all irradiated foods (Ehlermann, 2016; Eustice, 2018). It is noteworthy that this conclusion is based on the results of more than 30 years of health and safety research in various countries. In 1980, the Codex Alimentarius Commission (CAC) of FAO and WHO officially issued the “General Regulations for Irradiated Foods,” which provided the foundation for the hygiene and regulation of irradiated foods in various countries.

China officially joined the International Atomic Energy Agency (IAEA) in 1984 and became a member of the International Consultative Group on Food Irradiation (ICGFI) in 1994. In 1986, the Ministry of Health of China (MHC) promulgated the Interim Regulations on the Hygiene Management of Irradiated Foods, which approved the hygienic standards of 18 irradiated foods in three categories, including irradiated pollen, pork, and sweet potato wine (Anonymous, 1994.). In 1996, the “Interim Regulations on the Hygiene Management of Irradiated Foods” was abolished, and on April 5, 1996, the “Regulations of the National Health and Health Commission No. 47: Measures for the Hygiene Management of Irradiated Foods (MHMIF)” was promulgated. These regulations included general requirements for the personnel management system, licensing, employment, properties of irradiation facilities, management of irradiated food and maximum radiation dose (10 kGy), labeling, radiation processing of food, supervision, inspection, and other aspects (Anonymous, 1996). According to Article 19, irradiated food must be labeled on the packaging with a unified marking designed by the National Health Commission, establishing the framework for the management of irradiated food. In 1997, hygienic standards for five categories of irradiated food were reapproved in accordance with the recommendations of the ICGFI (GB1489.1‐GB1489.8) (Anonymous, 1997). In 2005, the MHC issued Decree No. 78, deciding to abolish the MHMIF. According to article 2, the state implements a licensing system for food irradiation processing. By 2001, a total of 18 technological standards for irradiated food were promulgated (Anonymous, 2001). From 2006 to 2013, there were 14 industrial standards for food irradiation (NY/T 1206–2006, etc.), and 25 import and export inspection and quarantine industry standards (SN/T 1887–2007, etc.) were successively promulgated to strengthen the management of the import and export of irradiated food. Additionally, 12 international standards were also promulgated successively, such as the International Recommended Guidelines for Radiation Processing of Food (CODEX STAN 106–1983, Rev.1–2003), International Recommended Practice for radiation‐processed foods (CAC/RCP 19–1979, Rev.2–2003), International Recommended Guidelines for Radiation Processing of Food (CAC/RCP 19–1979, Rev.1–2003), etc.

In 2008, the guidelines for phytosanitary measures and the standards for the detection and identification of radiation‐treated foods were promulgated (GB/T 21659–2008) (Anonymous, 2008). In 2009, the DNA comet test screening method for the identification of irradiated foods (GB/T 23748–2009) was enacted (Anonymous, 2009). In 2016, the national food safety standard “Hygienic Specification for Food Radiation Processing” (GB 18524) was released. According to Article 3.5 of the standard, the types of irradiated foods should be within the eight categories of products specified in GB 14891 (GB 14891.1–GB 14891.8), and other foods shall not be irradiated (Anonymous, 2016), as shown in Table 1. Among the above standards, GB is mandatory, and GB/T is recommended. Article 23 of the Food Safety Law (2019) stipulates that food irradiation processing shall comply with national food safety standards, and irradiated processed food shall be inspected and labeled in accordance with the requirements of national food safety standards (Anonymous, 2019).

**TABLE 1:** Health standards for food irradiation in China (Anonymous, 1996).

	List of foods allowed to be irradiated	Maximum absorbed dose limit
1	Cooked animal meat (pork, beef, rabbit, chicken, duck)	≤8 kGy
2	Pure pollen and mixed pollen of nectar source of corn, cultivated wheat, sorghum, sesame, rape, sunflower, and purple cloud.	≤8 kGy
3	Peanut kernel, longan, hollow nut, walnut, raw almond, red date, preserved peach, apricot, hawthorn, and other honey food	0.4–1 kGy
4	Anise, Chinese prickly ash, five‐spice powder, and other spices	≤10 kGy
5	Fresh fruits and vegetables (potato, onion, garlic, ginger, tomato, winter bamboo shoot, carrot, mushroom, sword bean, cauliflower, cabbage, water bamboo, apple, lychee, grape, kiwi, and strawberry)	Tomato ≤ 0.1 kGy Apple, Lychee, Kiwi ≤ 0.5 kGy Mushroom, Grape ≤ 1 kGy Strawberry ≤ 1.5 kGy Others ≤ 0.1 kGy
6	Frozen packaged meat of livestock and poultry (pig, cattle, sheep, chicken, duck, etc.)	≤2.5 kGy
7	Hog carcass	≤0.65 kGy
8	Beans, grains, and their products	Beans ≤ 0.2 kGy 0.4 kGy ≤ Grains ≤ 0.6 kGy

## 3 SCALE AND DISTRIBUTION OF RADIATION FACILITIES

China's first food to be irradiated on an industrial scale was dried potato wine (accelerated aging, de‐astringency, and flavor enhancement). Since 1984, China began to irradiate garlic, potatoes, onions, sweet potato wine, and meat products. In 1985, China introduced irradiated garlic into the Chinese food market for the first time in Henan province, and since then, there have been significant economic and social benefits achieved through the irradiation of condiments, functional foods, health foods, and recent aquatic products. As a result, numerous commercial irradiation devices have been established successively throughout China, distributed in more than 40 cities in 24 provinces, municipalities, and autonomous regions, such as Qingdao, Wuhan, Ningbo, Nanjing, Shanghai, Shenzhen, Beijing, and other large‐scale irradiation centers, which all are built for the irradiation of food (Chen, 2004).

**FIGURE 1:**
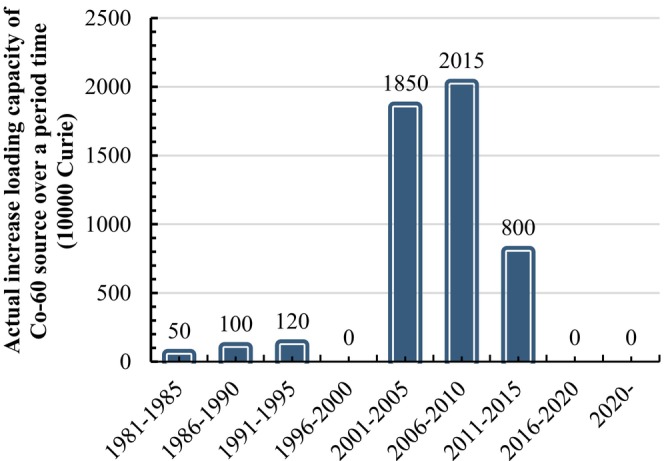
The increment source capacity of Co‐60 irradiation facility varies from year to year.

**FIGURE 2:**
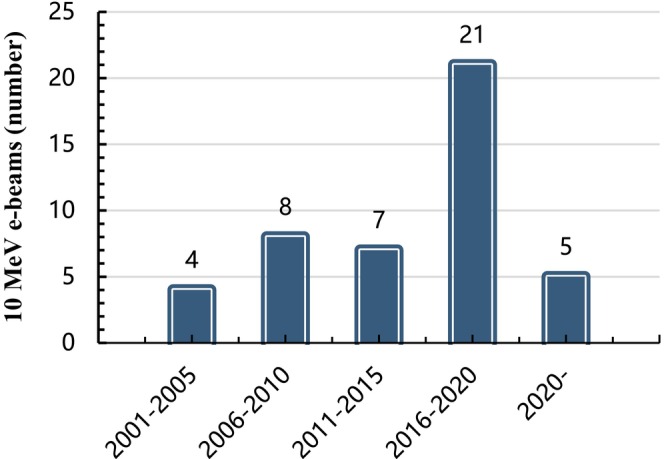
The increment number of electron accelerators varies from year to year.

At the end of 2019, there were over 120 γ‐irradiation installations in China with a design capacity of over 300 kCi, totaling ~176 MCi. Additionally, there were more than 50 10‐MeV high‐energy e‐beams in operation or under construction, with a total power of around 1, 200 kW. In 2016, the estimated total amount of food irradiation in China was close to 1 million tons per year (Ic & Cetinkaya, 2021). According to incomplete statistics, the total actual capacity of γ‐irradiation installations used for food irradiation is about 49.35 MCi (actual loading is 70 MCi, accounting for 23% of the world's total) with a total of 45 10 MeV e‐beams expected to be completed by the end of 2022, as shown in Figure 1 (Ic & Cetinkaya, 2021). It is worth noting that there has been zero increment capacity of Co‐60 source from 2010 to the present. Currently, companies selling irradiation facilities in China mainly include China Guangdong Nuclear Power Group Co. (CGNPC), China National Nuclear Corporation (CNNC), Beijing Genomics Institute (BGI), Leida Irradiation, Lanfu Irradiation, and China Gold Irradiation. As can be deduced from Figure 1, 80% of Co‐60 source irradiation facilities in China were built before 2010, while there were no new constructions after 2016, the increment was basically focused on the original design capacity. Furthermore, it can be deduced from Figure 2 that 75% of 10 MeV e‐beams were built after 2010, and the future growth of e‐beam facilities is expected to increase further in China. Three reasons are driving this trend. Firstly, the total cost of a 10 MeV 10 kW electron linear accelerator is estimated to be around 13–15 million yuan, which represents only 40%–60% of the cost of Co‐60 sources with equivalent irradiation processing capacity. Additionally, the review and approval process for the electronic linear accelerator project is known for its simplicity, speed, and short duration, with construction typically only about 1.5 years. Secondly, electron linear accelerators incur minimal expense when not in operation, as opposed to Co‐60 sources which continue to decay and require ongoing maintenance costs regardless of usage. Thirdly, unlike Co‐60 sources, electron linear accelerators do not generate nuclear waste. The high cost associated with nuclear waste disposal for Co‐60 sources is a significant consideration. In the event of a company going bankrupt, the burden of disposing of nuclear waste falls upon them at a considerable cost. It is noteworthy that, as of 2020, only five electron linear accelerators have been documented; there is a lack of statistics available for those that are planned or currently under construction.

**FIGURE 3:**
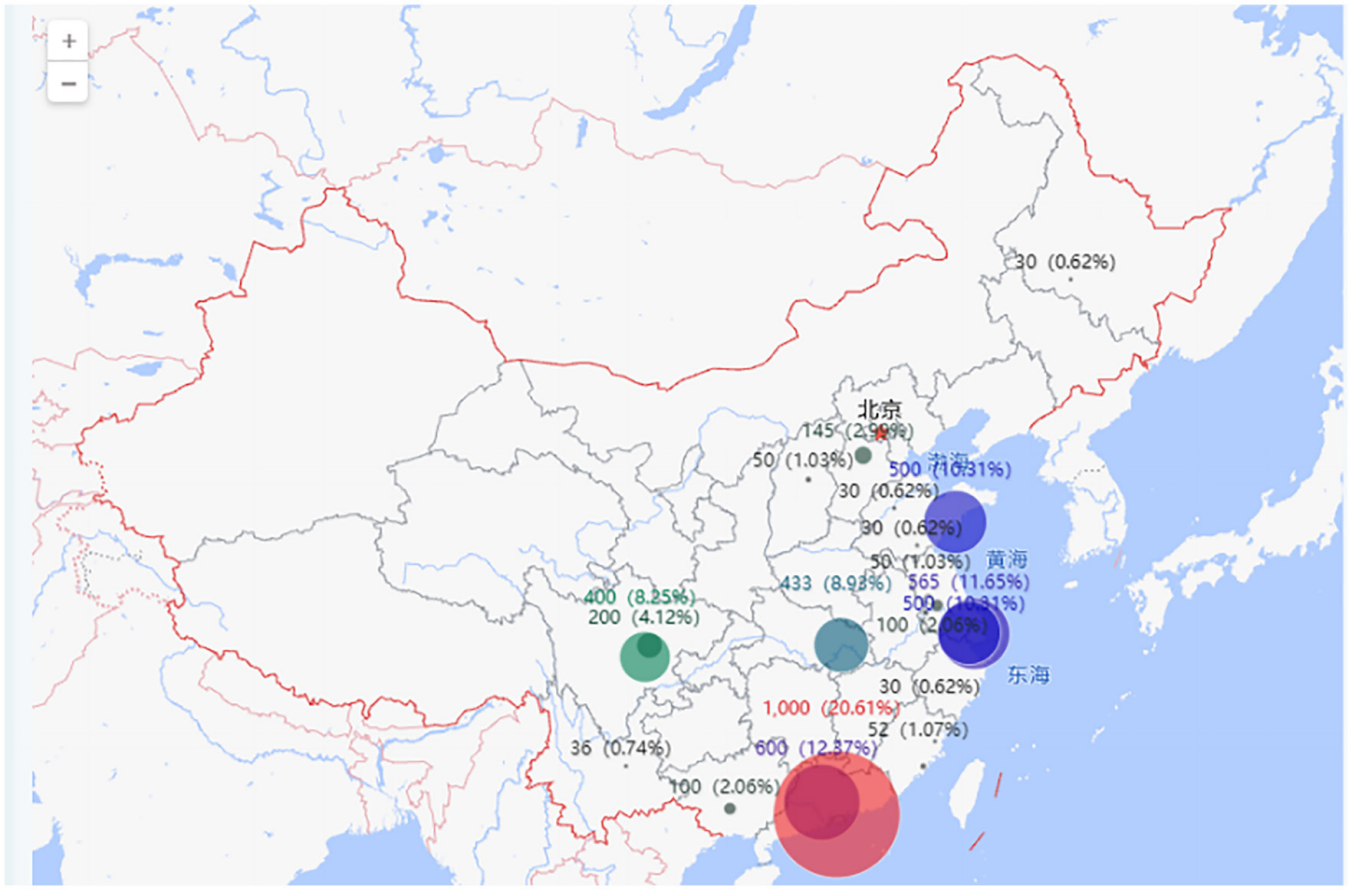
Geographical distribution of Co‐60 irradiation facilities in China.

**FIGURE 4:**
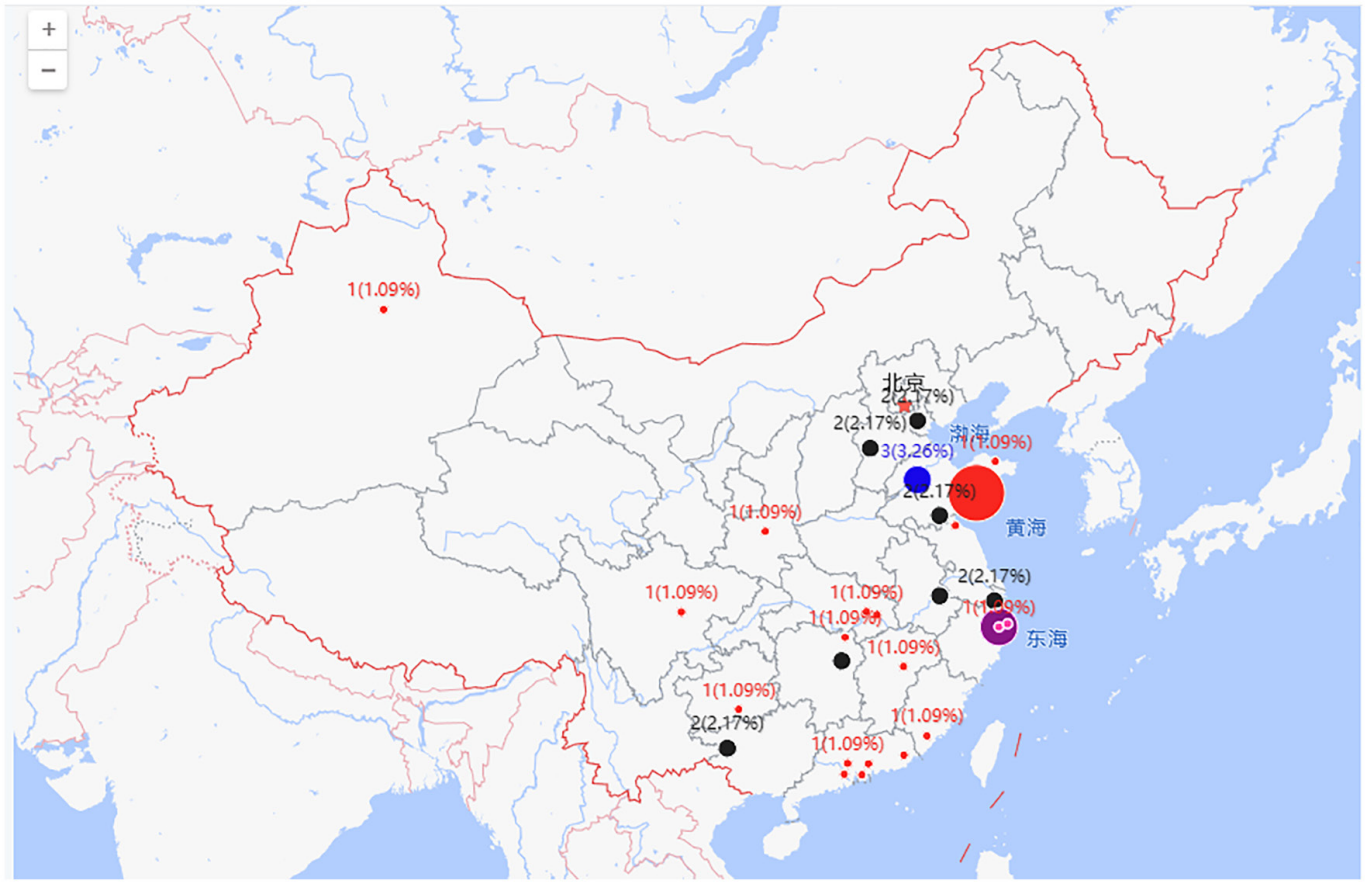
Geographical distribution of 10 MeV e‐beams in China.

In terms of geographical distribution, Co‐60 irradiation facilities are primarily concentrated in large coastal cities such as Bohai Bay, Yangtze River Delta, and Greater Bay Area, and Sichuan and Hubei provinces account for ~20% of the total facilities, mainly in the provincial capitals Chengdu and Wuhan. On the other hand, 10 MeV e‐beam facilities are mainly located in Bohai Bay and Yangtze River Delta regions and have gradually expanded to inland and border cities. These facilities are mainly used for border quarantine and sterilization of agricultural products to facilitate the import and export of agricultural products. The actual quantity of facilities is represented outside the parentheses, while the percentage of the total quantity is represented inside the parentheses, as shown in Figures 3 and 4.

## 4 CONSUMER ATTITUDES TOWARD IRRADIATED FOODS

The term “irradiation technology” has a large impact on the acceptability of the technology. When it comes to “food irradiation,” it often invokes negative associations such as nuclear power plants, genetic mutations, cancer, or radioactive food (Frewer et al., 2011; Jaeger et al., 2015). There is a lack of trust in the food industry, particularly in food processing technologies that consumers perceive as risky. As China is currently the fastest‐growing country in the world in food irradiation and also has the highest quantity of irradiated food, it is necessary to assess consumers' understanding of irradiated food and analyze the factors that affect their acceptance. To effectively address consumer concerns about irradiated food, it is essential to have a comprehensive understanding of not only their attitudes but also their awareness and acceptance. While previous studies have investigated consumer attitudes toward irradiated foods to some extent, our study aims to investigate these factors comprehensively, and also guide relevant departments in conducting science education to provide a guarantee for the sustainable development of irradiated food.

### 4.1 Materials and methods

In Hubei (China), a total of 290 questionnaires were collected, of which 264 questionnaires were deemed valid for analysis, while 26 research questionnaires were found to be invalid. The population characteristics used in these assessments mainly include gender, education level, age, and occupation, and eight inquiries (questions) about irradiated foods as shown in Table 2. The questionnaires were primarily distributed through WeChat and QQ, which are Tencent's two free instant messaging apps for smart devices.

**TABLE 2:** Validity analysis of questionnaire questions.

Case	Question
Case 1	Do you understand irradiated food?
Case 2	Have you ever seen a product/food with an irradiated label in the supermarket?
Case 3	Do you think irradiated food will harm human health?
Case 4	If you stumbled across the item you picked was irradiated food, would you continue to buy it?
Case 5	Can you accept that the food you eat is irradiated?
Case 6	Do you want to learn more about irradiated food?
Case 7	If authorities and scientific research show that irradiated food is safe, would you accept it?
Case 8	What is your general attitude towards irradiated food?

Quantitative analysis of the questionnaire was conducted using SPSS software, which included validity testing and correlation analysis. The correlation analyses involved univariate‐crossed chi‐square and bivariate‐crossed chi‐square to determine the strength and statistical significance of the association between any two classes of variables (Goldstein et al., 1976; Hosmane, 1986; The SPSSAU project, 2022).

### 4.2 Results analysis

#### 4.2.1 Description of consumer characteristics

Table 3 provides a description of population characteristics, including gender, age, education, and occupation. The sample consisted of 121 men and 143 women, with a small difference in gender ratio. In terms of age, the majority fell between 18 and 45 years old, with 18–25 years old accounting for 55.68% of the sample, indicating that the survey mainly targeted students, including undergraduates and graduate students. The age group of 25–45 years old accounted for 31%, while those under 18 and over 60 only accounted for 4.17% as verified by their academic background. Nearly 93% of the respondents had a university degree or above. In terms of occupation, the proportion of students is 54%, which aligns with the proportion of the 18–25 age group. Civil servants account for 10%, while full‐time staff such as teachers and doctors account for 25%.

**TABLE 3:** Frequency of population characteristics description.

Population characteristics	Option	Frequency	The percentage (%)
Gender	Male	121	45.83
	Female	143	54.17
Age	Under 18	8	3.03
	18 ~ 25	147	55.68
	25 ~ 45	82	31.06
	45 ~ 60	24	9.09
	Above 60	3	1.14
Education	Secondary school/technical secondary school and below	19	7.20
	University/Junior College	218	82.58
	Postgraduate or above	27	10.23
Occupation	Civil servants	27	10.23
	Professional staff (teachers/doctors/lawyers/athletes)	66	25.00
	Students	143	54.17
	Others	28	10.61
Total		264	100.0

#### 4.2.2 Validity analysis

Validity research is used to assess the reasonableness and meaningfulness of the research items. The data analysis method of factor analysis is used for validity analysis, and various indicators, such as KMO value, common degree, variance explanation rate value, and factor loading coefficient value, are comprehensively analyzed to determine the validity level of the data. The KMO value is used to judge the suitability of information extraction, the common degree is used to exclude unreasonable research items, the variance explanation rate value is used to describe the level of information extraction, and the factor loading coefficient is used to measure the corresponding relationship between factors (dimensions) and items. As shown in Table 4, the common degree values corresponding to all research items are higher than 0.4, indicating effective information extraction from the research items. In addition, the KMO value is 0.652, which is greater than 0.6, indicating that the data can be effectively extracted. The variance explanation rate values of the three factors are 25.019%, 18.906%, and 18.656%, respectively, with a cumulative variance explanation rate after rotation is 62.581% (>50%), which means that the information of the research item can be effectively extracted.

**TABLE 4:** Validity analysis of questionnaire questions.

	Factor loading coefficient			Common factor variance
	Factor 1	Factor 2	Factor 3	
Case 1	0.857	0.074	−0.062	0.744
Case 2	0.859	0.102	0.034	0.750
Case 3	−0.052	0.857	−0.128	0.754
Case 4	0.338	0.769	0.018	0.707
Case 5	−0.620	−0.070	0.157	0.414
Case 6	−0.044	0.185	0.702	0.529
Case 7	−0.019	−0.135	0.758	0.594
Case 8	−0.157	−0.335	0.615	0.515
Explain variance rate after (rotation)	25.019%	18.906%	18.656%	‐
KMO value	0.652			
*p*‐Value	.000			

#### 4.2.3 Correlation analysis

Correlation analysis is used to study the relationship between quantitative data, whether there is a relationship, and the degree of closeness of the relationship. Table 5 shows the correlation between the questionnaire questions. The statistical results of Case 1, Case 2, Case 4, and Case 6 show significant correlations with gender, with correlation coefficient values of −0.254, −0.379, −0.170, and −0.236, respectively. These values are all negative, indicating a negative correlation. Additionally, the statistical results of Case 4, Case 5, and Case 7 also show significant correlations with gender, with correlation coefficient values of −0.184, 0.175, and 0.216, respectively. In terms of education, only Case 1 and Case 5 show significant correlation, with a correlation coefficient value of 0.189. However, in terms of occupation, only Case 4 shows a significant correlation. This suggests that gender has the most prominent influence on consumers' attitudes toward irradiated food, followed by age, while the significance of education and occupation is less apparent.

**TABLE 5:** Relevance of demographic characteristics to survey questions.

	Gender	Age	Education	Occupation
Case 1	−.254**	−.062	.157*	−.003
Case 2	−.379**	−.120	.071	.095
Case 3	−.032	.019	−.038	.056
Case 4	−.170**	−.184**	.114	.189**
Case 5	.088	.175**	−.150*	−.020
Case 6	−.236**	−.074	−.001	.120
Case 7	.071	.216**	.002	−.088
Case 8	−.017	.086	.067	−.063

* *p* < .05.

** *p* < .01.

#### 4.2.4 Cross chi‐square analysis

Cross chi‐square analysis is employed to examine the relationship between quantitative data, determine the existence of a relationship, and assess the strength of association. In this study, crossed chi‐square is used to further explore the association between population characteristics and specific problems, as presented in Table 6. The results show that the proportion of women who have little knowledge about irradiated food is significantly higher at 54.55%, compared to men at 36.36%. Similarly, the proportion of women who had never paid attention to irradiated food is significantly higher at 84.62%, compared to men at 48.76%. In Case 3 (Do you think irradiated food is harmful to human health?), 13.99% of women chose “general risk,” which is significantly higher than men at 7.44%. Moreover, 26.57% of women choose “a bit dangerous,” significantly higher than men at 15.70%. These results indicate that women rarely know about irradiated food, mainly due to a lack of proactive attention to irradiated food. Partial knowledge about irradiation food leads to greater misunderstanding and lower acceptance.

**TABLE 6:** Results of crossover (Chi‐square) analysis of gender and survey questions.

Question	Answer options	Gender (%)		Total	*p*
		Male	Female		
Case 1	Know little	44 (36.36)	78 (54.55)	122 (46.21)	.000**
	Only heard	46 (38.02)	55 (38.46)	101 (38.26)	
	Understand better	31 (25.62)	10 (6.99)	41 (15.53)	
Case 2	I have not been paying attention	59 (48.76)	121 (84.62)	180 (68.18)	.000**
	No, because they rarely sell them	36 (29.75)	16 (11.19)	52 (19.70)	
	I have. I've seen it before	26 (21.49)	6 (4.20)	32 (12.12)	
Case 3	Very dangerous	28 (23.14)	30 (20.98)	58 (21.97)	.000**
	General hazard	9 (7.44)	20 (13.99)	29 (10.98)	
	A bit dangerous	19 (15.70)	38 (26.57)	57 (21.59)	
	Not dangerous	41 (33.88)	15 (10.49)	56 (21.21)	
	Not clear	24 (19.83)	40 (27.97)	64 (24.24)	
Case 4	Won't	40 (33.06)	68 (47.55)	108 (40.91)	.022*
	Unless there is a special need	32 (26.45)	38 (26.57)	70 (26.52)	
	If the harm is small, the price is cheap and the taste is good	49 (40.50)	37 (25.87)	86 (32.58)	
Case 5	Acceptable	61 (50.41)	50 (34.97)	111 (42.05)	.005**
	Unacceptable	17 (14.05)	42 (29.37)	59 (22.35)	
	Accept only knowingly	43 (35.54)	51 (35.66)	94 (35.61)	
Case 6	Want	95 (78.51)	135 (94.41)	230 (87.12)	.000**
	Do not want	26 (21.49)	8 (5.59)	34 (12.88)	
Case 8	Let consumers know about irradiated food and have the right to choose independently	93 (76.86)	117 (81.82)	210 (79.55)	.161
	State allows, security should be no problem	17 (14.05)	14 (9.79)	31 (11.74)	
	Radiation is horrible, better not to use it	9 (7.44)	5 (3.50)	14 (5.30)	
	Unsafe and should be banned	2 (1.65)	7 (4.90)	9 (3.41)	
Total		121	143	264	

* *p* < .05.

** *p* < .01.

For Case 5 (Can you accept that the food you eat is irradiated?), 50.41% of men chose “acceptable,” significantly higher than women at 34.97%. If informed the food they picked was irradiated food, 47.55% of women chose not to buy it, significantly higher than men at 33.06%. Among male consumers who chose “less harmful,” 40.50% of them selected the option of “if the price is cheap and the taste is good” significantly higher than female consumers at 25.87%. This indicates that half of men and one‐third of women are willing to accept that the food they have consumed is irradiated. However, if they are informed that they are buying irradiated food, their willingness to purchase is further reduced. This further shows that if consumers are informed about irradiated food through irradiation labels or other means without improving consumers' understanding of irradiated food, it may not be conducive to the sales of irradiated food.

For Case 6 (Do you want to know more about irradiated food?), 94.41% of women and 78.51% of men chose “yes” option. This result shows that consumers, in general, express a desire to learn more about irradiated food, indicating a limited availability of channels for consumers to obtain information on irradiated food, and the publicity of irradiated food needs to be further strengthened. For Case 8 (What is your general attitude towards irradiated food?). It can also be seen that 80% of consumers wish to strengthen publicity. Furthermore, only 3.41% of consumers believe that irradiated food is unsafe and should be banned, which is significantly lower than the 22% who do not accept it. That is to say, consumers who do not accept irradiated food are mainly concerned due to a lack of understanding, especially female consumers.

From Table 7, it can be observed that different age groups have varying levels of understanding and acceptance of irradiated food. Those under the age of 18 represent young people who have not yet graduated from high school; those aged 18–25 years are mainly college students, graduate students, and young people who have just started working; and those over 45 years represent middle‐aged and elderly people. Consumers in the 18–25 age group show higher understanding and acceptance of irradiated food. Notably, consumers of all age groups, except those in the 18–25 age group, believe that irradiated food is harmful and will not buy it. This is because consumers in the 18–25 age group prioritize taste and practicality more. On the other hand, consumers over the age of 45 have the lowest acceptance of irradiated food, with only 16.67%. However, 58% of consumers choose to accept it with knowledge, indicating that they are the main group that has concerns about irradiated food and requires more attention. It is also noteworthy that 100% of middle‐aged and elderly consumers express the desire to know more about irradiated food.

**TABLE 7:** Results of crossover (Chi‐square) analysis of age and survey questions.

Question	Answer options	Age (%)				Total	*p*
		Under 18	18 ~ 25	25 ~ 45	Above 45		
Case 1	Know little	5 (62.50)	62 (42.18)	41 (50.00)	14 (54.17)	122 (46.21)	.694
	Only heard	2 (25.00)	59 (40.14)	29 (35.37)	11 (41.67)	101 (38.26)	
	Understand better	1 (12.50)	26 (17.69)	12 (14.63)	2 (4.17)	41 (15.53)	
Case 2	I have not been paying attention	5 (62.50)	25 (17.01)	19 (23.17)	9 (29.17)	58 (21.97)	.094
	No, because they rarely sell them	0 (0.00)	20 (13.61)	8 (9.76)	1 (4.17)	29 (10.98)	
	I have. I've seen it before	0 (0.00)	35 (23.81)	16 (19.51)	6 (25.00)	57 (21.59)	
Case 3	Very dangerous	2 (25.00)	38 (25.85)	15 (18.29)	1 (4.17)	58 (21.97)	.037*
	General dangerous	1 (12.50)	29 (19.73)	24 (29.27)	10 (37.50)	29 (10.98)	
	A bit dangerous	5 (62.50)	45 (30.61)	42 (51.22)	16 (62.50)	57 (21.59)	
	Not dangerous	1 (12.50)	42 (28.57)	21 (25.61)	6 (20.83)	56 (21.21)	
	Not clear	2 (25.00)	60 (40.82)	19 (23.17)	5 (16.67)	64 (24.24)	
Case 4	Won't	4 (50.00)	75 (51.02)	27 (32.93)	5 (16.67)	108 (40.91)	.020**
	Unless there is a special need	2 (25.00)	22 (14.97)	28 (34.15)	7 (25.00)	70 (26.52)	
	If the harm is small, the price is cheap and the taste is good	2 (25.00)	50 (34.01)	27 (32.93)	15 (58.33)	86 (32.58)	
Case 5	Acceptable	4 (50.00)	75 (51.02)	27 (32.93)	4 (16.67)	111 (42.05)	.007**
	Unacceptable	2 (25.00)	22 (14.97)	28 (34.15)	6 (25.00)	59 (22.35)	
	Accept only knowingly	2 (25.00)	50 (34.01)	27 (32.93)	14 (58.33)	94 (35.61)	
Case 6	Want	6 (75.00)	125 (85.03)	74 (90.24)	25 (100.00)	230 (87.12)	.009**
	Do not want	2 (25.00)	22 (14.97)	8 (9.76)	2 (0.00)	34 (12.88)	
Total		8	147	82	27	264	

* *p* < .05.

** *p* < .01.

Furthermore, from an educational perspective, it can be observed that consumers with higher education levels tend to or have more knowledge about irradiated food, which is in accordance with the Table 7 analysis. As shown in Table 8, in relation to Case 2 (Do you think irradiated food is harmful to human health?), ~22% of consumers chose the option “very dangerous.” However, there was a sharp difference in the percentage of consumers who chose the option of “not dangerous,” 10.53% of consumers have a middle school degree or below, 19.3% have a college degree, and 44.4% have a graduate degree or above. On the other hand, in relation to Case 5 (Can you accept that the food you eat is irradiated?), over half (58%) of consumers with lower levels of education (Secondary school and below) stating that they can only accept it if they know about the irradiation beforehand. This was confirmed in the survey conducted in Case 4 (“If you stumbled across the item you picked was irradiated food, would you continue to buy it?”), if informed that they are purchasing irradiated food, 42.11% of consumers would choose to abandon their purchase. This suggests that low‐educated consumers do not accept irradiated food mainly due to a lack of knowledge about irradiated food, highlighting the need to strengthen popular science propaganda.

**TABLE 8:** Results of cross (chi‐square) analysis of educational background and survey questions.

Question	Answer options	Education (%)			Total	*p*
		Secondary school and below	College students	Graduate and above		
Case 1	Know little	97.(437)	105 (48.17)	8 (29.63)	122 (46.21)	.022*
	Only heard	10 (52.63)	81 (37.16)	10 (37.04)	101 (38.26)	
	Understand better	0 (0.00)	32 (14.68)	9 (33.33)	41 (15.53)	
Case 2	I have not been paying attention	12 (63.16)	153 (70.18)	15 (55.56)	180 (68.18)	.078
	No, because they rarely sell them	7 (36.84)	37 (16.97)	8 (29.63)	52 (19.70)	
	I have. I've seen it before	0 (0.00)	28 (12.84)	4 (14.81)	32 (12.12)	
Case 3	Very dangerous	4 (21.05)	47 (21.56)	7 (25.93)	58 (21.97)	
	General hazard	1 (5.26)	28 (12.84)	0 (0.00)	29 (10.98)	
	A bit dangerous	4 (21.05)	48 (22.02)	5 (18.52)	57 (21.59)	.027**
	Not dangerous	2 (10.53)	42 (19.27)	12 (44.44)	56 (21.21)	
	Not clear	8 (42.11)	53 (24.31)	3 (11.11)	64 (24.24)	
Case 4	Won't	8 (42.11)	91 (41.74)	9 (33.33)	108 (40.91)	.152
	Unless there is a special need	7 (36.84)	60 (27.52)	3 (11.11)	70 (26.52)	
	If the harm is small, the price is cheap and the taste is good	4 (21.05)	67 (30.73)	15 (55.56)	86 (32.58)	
Case 5	Acceptable	5 (26.32)	91 (41.74)	15 (55.56)	111 (42.05)	
	Unacceptable	3 (15.79)	50 (22.94)	6 (22.22)	59 (22.35)	
	Accept only knowingly	11 (57.89)	77 (35.32)	6 (22.22)	94 (35.61)	
Total		19	218	27	264	

* *p* < .05.

** *p* < .01.

## 5 DISCUSSION

Due to the increasing domestic demand, China is facing significant development pressure in terms of food supply and food safety. As a result, the variety and quantity of commercially irradiated food in China have gradually increased in recent decades, with China currently supplying over 50% of the world's irradiated food. The Chinese government has placed increased emphasis on the establishment of relevant policies, regulations, and regulatory systems to ensure and enforce microbiological safety, pest free, and long shelf life of irradiated food at all stages of food processing to meet consumer's needs under the food safety framework as a precautionary measure. With the breakthrough of electron accelerator technology in China, the number of e‐beams irradiation facilities is increasing annually. On the other hand, the construction of Co‐60 irradiation facilities has been declining annually with no trend toward construction in the future. These radiation facilities are mainly located in the Bohai Bay, Yangtze River Delta region, and the Greater Bay Area, however, these days the development is spreading into the inland, western and southern border cities.

Through this study, we have identified that Chinese consumers have relatively low awareness and knowledge of food irradiation, with ~84.5% of consumers knowing little or having only heard of irradiated food. It was identified that consumers' acceptance of irradiated food is directly proportional to their understanding: 42% of the sampled population indicated they could accept that they have eaten irradiated food, 35% of the sampled consumers indicated they will accept it if they knew it, and 22% of the consumers would not accept it. Although many consumers accept that they have eaten irradiated food, they will not take the initiative to buy irradiated food because of a lack of information about the risks and benefits and often even misinformation (Food safety news, 2010), as well as the negative associations caused by the label “irradiation” (Bearth & Siegrist, 2019). This phenomenon is more pronounced among female, low‐educated, and older consumers. The proportion of consumers who indicated they would not buy irradiated food under any circumstance was about 40%. However, it can be inferred in our analysis that if we strengthen the understanding of female, low‐educated, and older consumers of irradiated food, the overall consumer acceptance can be greatly improved (up to ~60%). Another study also found that positive attitudes toward irradiated food increased from 50 to 60% to 80–90% when people were trained in lecture training (Food safety news, 2010). Often, negative information has a greater impact, so it is crucial to educate the public to become more rational about irradiation food, and to educate policymakers and leaders on the reasons why routine use of food irradiation could be beneficial.

Although we have a general understanding of the relationship between consumers' characteristics and their attitudes toward irradiated food. However, it is currently unclear which factors have the greatest impact on consumers' attitudes towards irradiated food. It may be one or several of the following factors, such as fear of radiation (nuclear accidents and weapons), preference for a natural and green lifestyle, health concerns, innate resistance to new technologies, distrust of the unknown, or other factors. Further research is needed in the next step to investigate how these factors affect consumers' attitudes towards irradiated food.

## AUTHOR CONTRIBUTIONS

**Ke Wang:** Data curation (lead); investigation (lead); methodology (equal). **Xingxing Pang:** Data curation (equal); formal analysis (equal); investigation (equal). **Zhengkui Zeng:** Investigation (equal); methodology (lead); project administration (lead); resources (lead); supervision (lead); writing – review and editing (lead). **Houhua Xiong:** Writing – review and editing (equal). **Jifu Du:** Writing – review and editing (equal). **Gang Li:** Data curation (equal). **Isaac Kwasi Baidoo:** Supervision (supporting); writing – review and editing (lead).

## FUNDING INFORMATION

This work was supported by the Key Laboratory of Radiation Chemistry and Functional Materials of Hubei Province Grant No. 2022ZX06 and the Ph.D. Initiation Fund Project Grant No. BK202207.

## CONFLICT OF INTEREST STATEMENT

The authors declare that they have no financial conflict of interest or personal relationships that influence the work described on this page.

## ETHICS STATEMENT

This study involved no humans or animals.

## Data Availability

The data that support the findings of this study are available on request from the corresponding author. The data are not publicly available due to privacy or ethical restrictions.
